# Evolution of field induced magnetic phase attributed to higher order magnetic moments in TbVO_4_

**DOI:** 10.1038/s41598-023-27804-z

**Published:** 2023-01-10

**Authors:** Dheeraj Ranaut, K. Mukherjee

**Affiliations:** School of Physical Sciences, Indian Institute of Technology Mandi, Mandi, Himachal Pradesh 175075 India

**Keywords:** Condensed-matter physics, Condensed-matter physics

## Abstract

Study of quantum magnetism in rare earth orthovanadates (RVO_4_, R = rare earth) is a topic which is currently being investigated by the condensed matter physicists. In this work, through both experimental and theoretical tools, we report the presence of field induced magnetic phase, attributed to fifth order susceptibility, in TbVO_4_, at low temperatures. The structural transition reported around 31 K, results in the formation of pseudospin—$${\raise0.7ex\hbox{$1$} \!\mathord{\left/ {\vphantom {1 2}}\right.\kern-0pt} \!\lower0.7ex\hbox{$2$}}$$ doublet ground state separated by an energy *δ*. Temperature dependent heat capacity indicates toward an increment in *δ*, on application of magnetic fields. Above 10 kOe, the Zeeman energy associated with magnetic anisotropy strengthens, resulting in an enhanced splitting of the pseudo-doublet ground state. This increased splitting stabilizes the magnetic phase associated with higher order moments. These observations are further supported by our theoretical model to evaluate *δ*, as a function of applied field. Our study provides a platform to study the possible presence of higher order moments in other Jahn–Teller systems.

## Introduction

During the last decade, investigation of quantum magnetism in 3D spin systems has gained the interest among condensed matter physicists as these systems exhibit exotic magnetic ground states like spin ice^1,2^, quantum spin liquid (QSL)^3–5^ and low dimensional magnetism^6–8^. In this context, rare earth (R)-based series RVO_4_ is catching attention in recent times. Members of this family exhibit exotic magnetic ground states depending on different properties like strong crystal field effects, magnetic interactions corresponding to near neighbour R atoms and unusual easy-axis of magnetization. CeVO_4_ shows the evidences of strong crystal field effects which results in magnetic ground state associated with effective spin—$${\raise0.7ex\hbox{$1$} \!\mathord{\left/ {\vphantom {1 2}}\right.\kern-0pt} \!\lower0.7ex\hbox{$2$}}$$^9^. Another member HoVO_4_ exhibits signatures of QSL state due to the dominance of frustrated 2nd and 3rd near neighbour Ho atoms^10^. In ErVO_4_, a field induced magnetic anomaly along with a magnetic phase ascribed to positive fifth order susceptibility is reported^11^. The origin of this is believed to be unusually large magnetoelastic coupling along the easy c-axis of magnetization which is in contradiction to other members of the series. Along with this, TmVO_4_ reveals magnetic field tuned ferroquadrupolar quantum phase transition^12,13^ and anisotropic nematic fluctuations above this ferroquadruople transition^14^. Investigations on DyVO_4_ divulge the presence of field tuned quantum criticality^15^. As far as structural, thermodynamic and optical properties are considered, this series had been studied through various experimental tools in last few decades. The compounds of this series crystallize in the tetragonal Zircon structure with space group $$D_{4h}^{19}$$ or *I*4_1_/amd at room temperature^16^. They exhibit interesting magnetic and optical properties due to the presence of indirect super-exchange interactions and 4*f* electron–phonon coupling^17–21^. The magnetic R^3+^ ion in RVO_4_ is expected to unveil magnetic ordering at very low temperatures. Along with this, some of the compounds also undergo structural transition to a lower symmetry, induced by the cooperative Jahn–Teller (JT) effect^19,21^. This structural transition is expected to bring significant changes in the crystal field levels in the low symmetry phase of the respective compounds. Hence in order to explore the effect of magnetic field on these levels, TbVO_4_, which exhibit JT driven structural transition, can be an interesting candidate.

**Figure 1:**
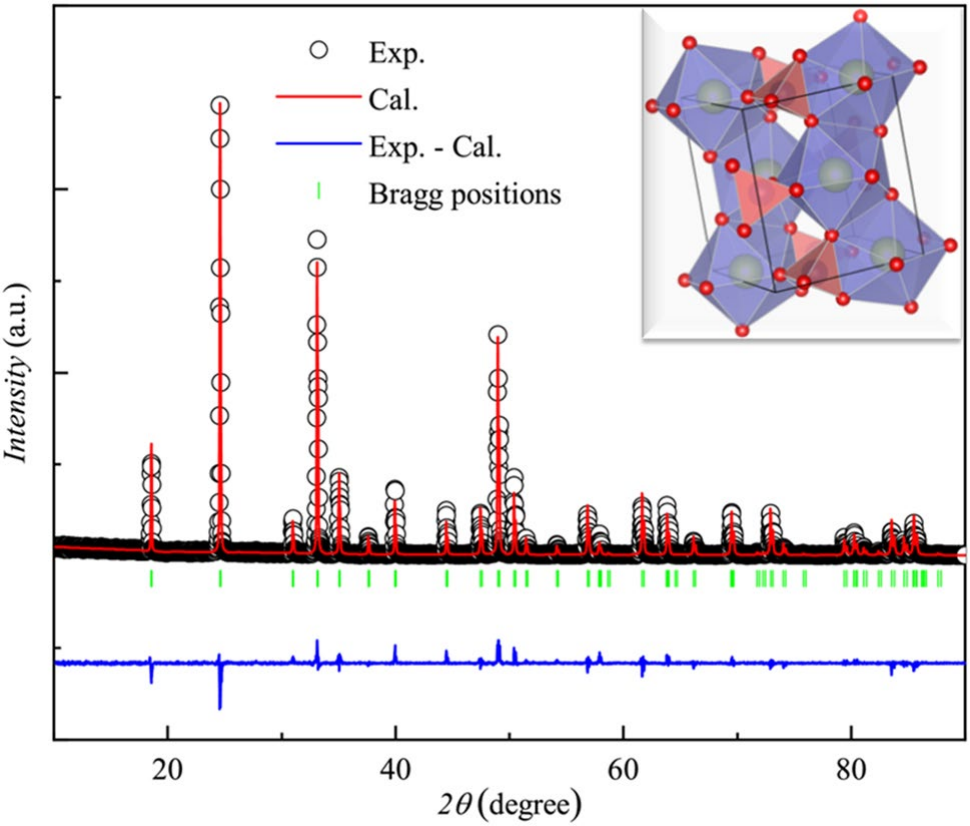
Rietveld refined powder-XRD pattern of TbVO_4_ obtained at room temperature. The black open circles indicate the experimental data, while the Rietveld refinement fit is shown as solid red line. The solid blue line and green vertical lines show the difference curve and Bragg positions, respectively. The inset shows its crystal structure.

TbVO_4_ undergoes a structural transition from the higher symmetric tetragonal phase to a lower symmetric orthorhombic phase with space group Fddd ($$D_{2h}^{24}$$) at around *T*_*D*_ ~ 33 K^22^. Raman and optical studies on this compound reveal that the transition is of second order and is associated with an orthorhombic distortion of B_2g_ symmetry^23,24^. Temperature dependent X-ray diffraction studies reveal that below *T*_*D*_, the observed changes in the lattice parameters are very small^25^. However, below *T*_*D*_, the crystal field energy levels exhibit significant difference from the tetragonal phase because of the JT effect. Tb^3+^ (4f^8^, ^7^F_6_) is a non-Kramer ion. In the higher symmetry phase, due to tetragonal crystal field effect, the ^7^F_6_ manifold is splitted into three doublets and seven singlets^26^. The first four energy levels are comprised of a ground state singlet (Γ_1_); the next level is a non-Kramer doublet (Γ_5_) at about 12 K, followed by another singlet (Γ_3_) at 27.5 K^27,28^. Below *T*_*D*_, a noteworthy change in the energy levels is observed as a consequence of JT effect^26–29^. The Γ_3_ singlet is raised in energy to 73.8 K from 27.5 K, transforming into a Γ_1_ singlet. The Γ_5_ doublet is splitted into two components, one of which is raised in energy by 67.6 K and other is reduced, lying just above the ground state Γ_1_ singlet defined in the tetragonal phase. This energy level scheme is very well defined in the Fig. 1 of the reference^26^. These four energy levels are believed to solely determine the properties of TbVO_4_ below *T*_*D*_. The two lowest singlets are treated as a non-Kramer pseudo-doublet with *S* = $${\raise0.7ex\hbox{$1$} \!\mathord{\left/ {\vphantom {1 2}}\right.\kern-0pt} \!\lower0.7ex\hbox{$2$}}$$, having a splitting of *δ* ~ 1.3 K^29^. Even though, TbVO_4_ had been studied by various experimental techniques, but to the best of our knowledge, no attempts were made to study the effect of magnetic field on the splitting and its resulting consequences.

Hence, in this work, we have focused on the following queries: whether on application of magnetic field (i) the splitting *δ* increases; (ii) is there any magnetic phase formation associated with higher order moments. Our studies reveal that field dependent magnetization at 2 K shows the hint of evolution of a magnetic phase above 10 kOe. Temperature dependent heat capacity indicates towards the increment in *δ* on applying external magnetic field. Non-linear DC susceptibility measurements show the development of higher order moments above 10 kOe which are associated with fifth order susceptibility (*χ*_5_). It is noted that above 10 kOe, Zeeman energy related with magnetic anisotropy plays an imperative role and results in the increased splitting of the pseudospin—$${\raise0.7ex\hbox{$1$} \!\mathord{\left/ {\vphantom {1 2}}\right.\kern-0pt} \!\lower0.7ex\hbox{$2$}}$$ doublet ground state. This increased splitting is believed to give rise to the development of positive *χ*_*5*_ at low temperatures and higher fields. To further support our experimental observation, we have used a theoretical model to calculate *δ* as a function of magnetic field. It is found that *δ* increases as the field increases. Our investigations also indicate towards an increased splitting of the pseudo-doublet ground state on application of magnetic field, which further stabilizes the interaction among higher order moments.

## Results

### Crystal structure

The Rietveld refined pattern of the powder X-ray diffraction (XRD) data shows that TbVO_4_ crystallizes in single tetragonal phase with space group *I*4_1_/amd (141) (shown in the Fig. 1). The obtained lattice parameters a = b = (7.176 ± 0.004) Å, c = (6.326 ± 0.004) Å and volume = (325.76 ± 0.030) Å are found to be in good agreement with the previous reports^16^. The inset of Fig. 1 depicts the crystal structure of TbVO_4_. It is comprised of isolated VO_4_ tetrahedron (orange color) which share corners and edges with TbO_8_ bisdisphenoids (purple color). Each Tb atom is bonded with four different O atoms at two different distances in the form of compressed and elongated bonds.

### Magnetization

**Figure 2:**
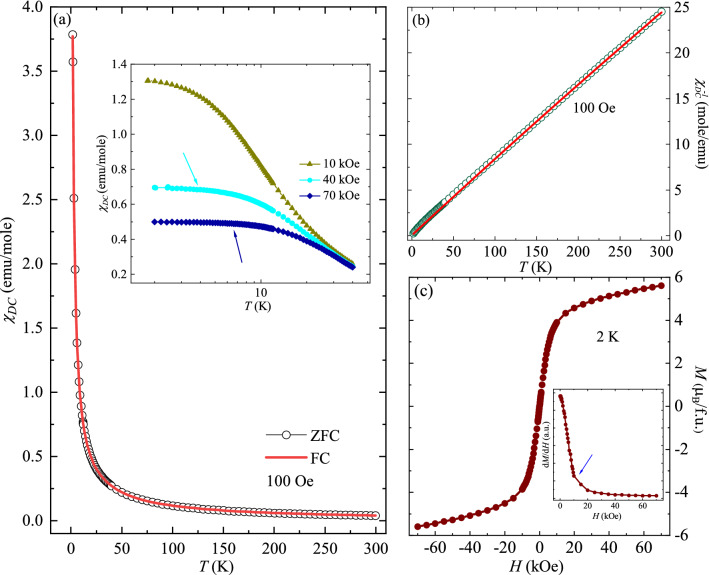
(**a**) *χ*_*DC*_ as a function of *T* measured at 100 Oe under ZFC and FC protocols. The inset shows the same quantity measured at various applied *H* up to 70 kOe on the log scale. The arrows denote the onset of the saturation. (**b**) *T* response of $\chi_{DC}^{ - 1}$ at 100 Oe field. The solid red line denotes the fitting to the CW law (1). (**c**) Isothermal magnetization curve obtained at 2 K. Inset: *H* dependence of the derivative of the magnetization with respect to field at 2 K. The arrow indicates the slope change around 10 kOe.

Figure 2a depicts the temperature (*T*) dependent DC magnetic susceptibility (*χ*_*DC*_) measured under zero field cooling (ZFC) and field cooling (FC) protocols at 100 Oe in the range 1.8–300 K. It is observed that on decreasing the *T* below 50 K, *χ*_*DC*_ continuously increases and no signature of magnetic ordering is seen up to 1.8 K. Along with this, both ZFC and FC curves follow identical path. Further, *T* response of *χ*_*DC*_ under ZFC protocol at various applied *H* up to 70 kOe is also measured and is shown in the inset of Fig. 2a. On increasing the *H*, *χ*_*DC*_ starts saturating, signifying that the Tb^3+^ spins start aligning along the direction of applied *H*. *χ*_*DC*_ seems to saturate below 4 K and 6.6 K for 40 kOe and 70 kOe curves, respectively (shown by the arrows). The change in behaviour of *χ*_*DC*_ as a response to applied *H* indicates that the magnetic field alters the interaction among the Tb^3+^ spins. The *T* dependent inverse DC magnetic susceptibility ($\chi_{DC}^{ - 1}$) at 100 Oe field is plotted in the Fig. 2b. The data is fitted with Curie–Weiss (CW) law using the relation:

1 $$\chi_{DC} = \chi_{0} + \frac{C}{{T - \theta_{CW} }}$$

here, *C* is the Curie constant and *Ɵ*_*CW*_ is CW temperature. The term *χ*_*0*_ contains the contribution from both core diamagnetic susceptibility and *T* independent Van Vleck paramagnetic susceptibility. From Fig. 2b, it is noted that the curve is well fitted at higher temperatures and a non-linear deviation is observed below 50 K. This observed deviation can be attributed to crystal field effects (CFE). The obtained values of *χ*_*0*_, *C* and *Ɵ*_*CW*_ are ~ (1.67 × 10^–3^ ± 0.17 × 10^–3^) emu/mole-Oe, ~ (11.79 $\pm$ 0.08) emu/mol-Oe-K and ~—(5.44 $\pm$ 0.18) K, respectively. The experimentally obtained effective magnetic moment $\mu_{eff}$ (= 2.83 $\sqrt C \mu_{B}$ ~ 9.71 $\mu_{B}$), matches very well with the theoretically calculated $\mu_{eff}$ = 9.72 $\mu_{B}$ for Tb^3+^ [calculated using relation: $\mu_{eff}$ = *g* $\sqrt {J \left( {J + 1} \right)}$ $\mu_{B}$, where *g* = 1.5, *S* = 3, *L* = 3, *J* = 6]. The negative value of *θ*_*CW*_ implies the presence of antiferromagnetic (AFM) correlations among the Tb^3+^ spins. Figure 2c represents the isothermal magnetization, *M* (*H*) curve obtained at 2 K. It is observed that magnetization (*M*) increases linearly with *H* up to 10 kOe and then saturation starts on further increasing the field. It reaches a value of ~ 5.6 µ_B_/Tb^3+^ at 70 kOe field which corresponds to about 58% of the theoretically calculated value of 9.72 µ_B_ for Tb^3+^ free ion. Generally, in polycrystalline samples, relatively lower value of the magnetization at such high field, as compared to the theoretically calculated free moment of the magnetic ion, is a signature of presence of magnetic anisotropy^15,30^. This is found to be in accordance with the earlier reports on single crystals of TbVO_4_^31^, where TbVO_4_ is reported to possess strong magnetic anisotropy along a-axis (easy axis). In addition, a change in slope is observed around 10 kOe, shown by the arrow in the inset of Fig. 2c, displaying d*M*/d*H* as a function of *H*. The next section focuses on the *T* dependence of heat capacity at various applied *H*, as it can provide further information on the magnetic ground state.

### Heat capacity

**Figure 3:**
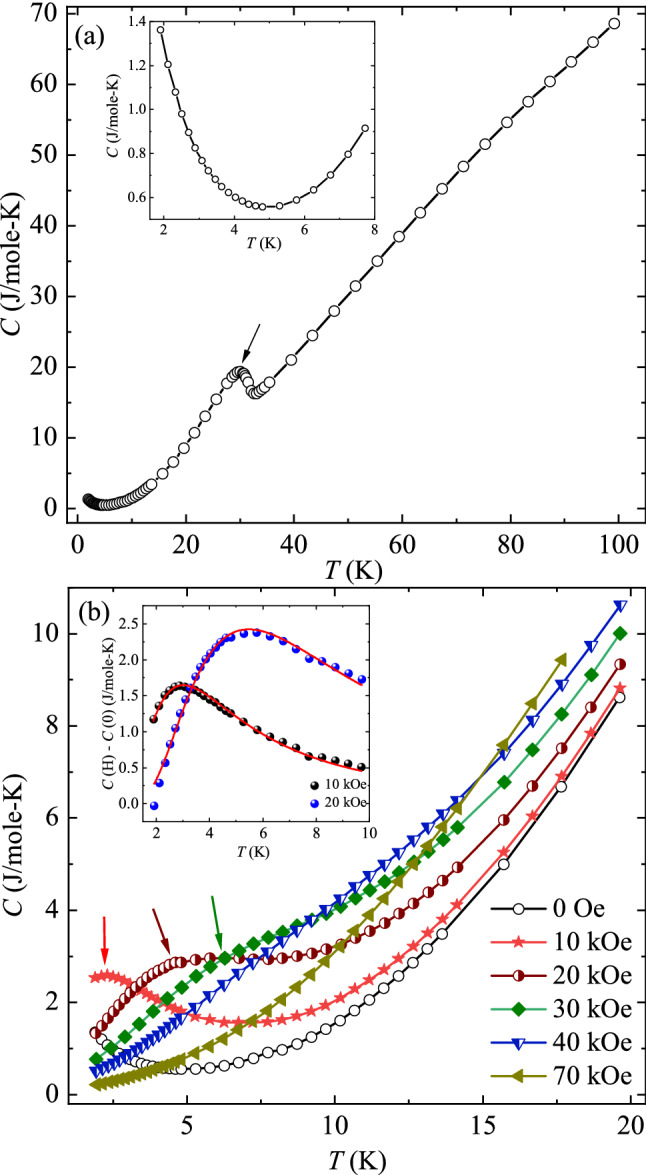
(**a**) *T* dependent *C* measured at zero field, with arrow showing the temperature of structural transition. The inset shows the same quantity below 10 K. (**b**) *C* versus *T* curves obtained at various applied *H*. The arrows show the emergence of broad hump. Inset: Two-level Schottky fitting obtained at 10 and 20 kOe curves.

Heat capacity (*C*) acts as a sensitive probe to get an insight about the ground state and low energy excitations between the ground and excited states. So, in order to get an understanding of the effect of externally applied *H* on the magnetic ground state of TbVO_4_, we have measured the *T* dependent *C* in the presence of *H* up to 70 kOe. Figure 3a shows the *T* dependent *C* measured at zero field in the range of 1.8–100 K. The curve is manifested by the presence of an anomaly around 31 K (shown by the black arrow), followed by an upturn at lower temperature. The high *T* anomaly arises due to the presence of structural transition from the high symmetric tetragonal phase to the low symmetry orthorhombic phase. In accordance to previous reports, this structural transition is attributed to JT effect which is further believed to bring significant changes in the crystal field energy levels in the orthorhombic phase^27–29^. The observed anomaly at the temperature ~ 31 K, matches very well with previous literature reports^22,32^. Further, to clearly visualize the low temperature upturn, we have plotted *T* dependent *C* at 100 Oe below 8 K in the inset of Fig. 3a. As noted from the figure, *C* shows an upturn around 5 K and starts increasing on further decreasing the temperature. As mentioned above, in our system TbVO_4_, the crystal field scheme is drastically affected by the JT distortion below the transition temperature^26–29^. In the low symmetry orthorhombic phase, the lowest two singlets are treated as pseudospin—$${\raise0.7ex\hbox{$1$} \!\mathord{\left/ {\vphantom {1 2}}\right.\kern-0pt} \!\lower0.7ex\hbox{$2$}}$$ doublet, separated by an energy difference of δ ~ 1.3 K^29^. So, the observed low temperature feature is believed to originate from the Schottky anomaly arising due to the excitation between the levels of pseudo-doublet state. Further, in order to understand the effect of externally applied *H*, *T* dependent *C* in the presence of different *H* is shown in the Fig. 3b. As visible from the figure, the 10 kOe curve exhibit a weak hump around 2.3 K which shifts to 4.4 K for 20 kOe (shown by the arrows). On further increasing the *H*, the hump broadens and shifts to higher *T*. This hump vanishes completely for 70 kOe curve. This implies that at zero field, the hump is situated below 1.8 K and shifts to higher *T* on application of external *H*. This observed broad hump in *C* is generally reported in systems having doublet ground state. Its broadening at higher fields takes place due to the increased splitting of the doublet state as a consequence of Zeeman effect^33^. Thus, the observed low temperature upturn in *C* at zero field is believed to arise due to the presence of pseudo-doublet ground state with a splitting of *δ*. Shifting of the hump to higher temperatures on applying the external *H* hint towards the increment of *δ* as a response to the *H.*

In order to shed more light on the *H* dependence of the splitting *δ*, we have used two-level Schottky scheme given as^9^:

2 $$C(H{\text{kOe}}){-}C\left( {0{\text{ kOe}}} \right) = N_{A} k_{B} \frac{{\left( {\delta /T} \right)^{2} e^{ - \delta /T} }}{{\left( {1 + e^{ - \delta /T} } \right)^{2} }}$$

Here, *N*_*A*_ and *k*_*B*_ are Avogadro’s number and Boltzmann constant, respectively. *δ*, as mentioned above, describes the splitting between the pseudospin—$${\raise0.7ex\hbox{$1$} \!\mathord{\left/ {\vphantom {1 2}}\right.\kern-0pt} \!\lower0.7ex\hbox{$2$}}$$ doublet ground state. The inset of Fig. 3b depicts the *T* response of (*C* (*H*) − *C* (0)) curves at 10 and 20 kOe, along with solid red lines showing the fit to the Eq. (2). The obtained value of *δ* for 10 kOe and 20 kOe curves is (7.22 ± 0.04) K and (13.13 ± 0.11) K, respectively. This implies that the splitting increases on application of external *H*. Thus, the observed shifting of hump in *C* to higher *T* along with the increment in the value of *δ* obtained from the fitting signifies the presence of enhanced splitting of pseudo spin—$${\raise0.7ex\hbox{$1$} \!\mathord{\left/ {\vphantom {1 2}}\right.\kern-0pt} \!\lower0.7ex\hbox{$2$}}$$ doublet ground state with *H*. The observed behaviour in *C* at low temperatures gives rise to a query that whether this increased splitting can give rise to another field induced magnetic phase, different from the low/zero field magnetic state. Thus, in the next section, we have discussed non-linear DC susceptibility to investigate the possible presence of a magnetic phase associated with higher order moments.

### Non-linear DC susceptibility

**Figure 4:**
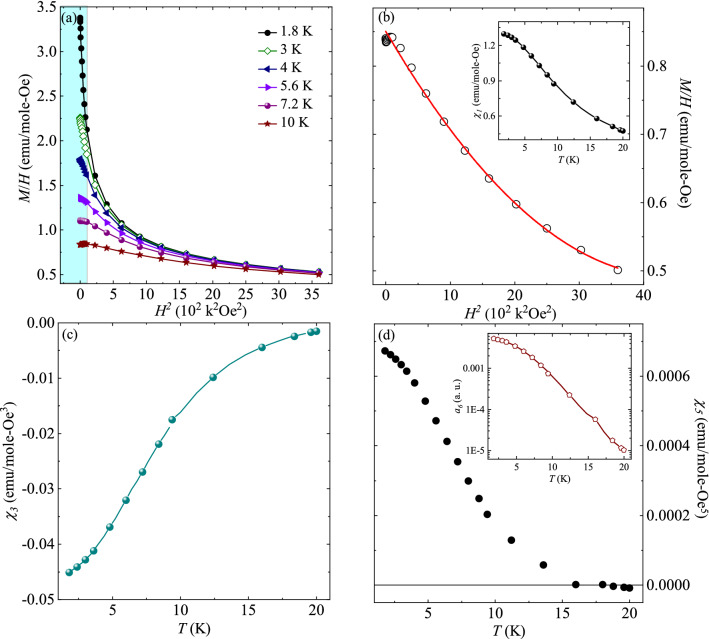
(**a**) *M*/*H* vs *H*^*2*^ curves plotted at various temperatures. The shaded region below 10 kOe shows the deviation from quadratic-like behaviour. (**b**) *M*/*H* versus *H*^2^ curve at 10 K, with solid red line showing the fit to the Eq. (3). The inset of (**b**) shows the temperature response of linear term *χ*_1_. (**c**, **d**) Temperature response of *χ*_3_ and *χ*_5_ extracted from the non-linear DC susceptibility data. The inset of (**d**) shows the quantity ${\text{a}}_{{6}} \left\{ { = [{3}\chi_{3}^{2} - \chi_{5} \chi_{1} ]/\chi_{1}^{7} } \right\}$ plotted as a function of temperature.

Non-linear DC susceptibility acts as an important tool to probe the nature of exotic spin ordering and to investigate the presence of higher order moments. Generally, magnetization along the direction of magnetic field can be expanded in the form^34–38^:

3 $$M/H = \chi_{1} + \chi_{3} H^{{2}} + \chi_{5} H^{{4}}$$

where *χ*_1_ is the linear term associated with dipolar moments, *χ*_3_ and *χ*_5_ are the non-linear terms associated with higher-order moments. In order to evaluate the higher order moments (*χ*_3_ and *χ*_5_), magnetization data was collected under the FC condition with slow cooling rate of 5 mK/s at a constant field, similar to the protocols mentioned in the references^35,38^. *M*/*H* versus *H*^2^ curves at different temperatures are plotted in the Fig. 4a. From the figure, it is clearly visible that *M*/*H* versus *H*^2^ curves follow quadratic-like behaviour above 10 kOe and then a deviation is observed, as field is lowered below 10 kOe (shown by the shaded region). In the quadratic-like region 10–60 kOe, the fit to the Eq. (3) yields the non-linear terms (*χ*_3_ and *χ*_5_) with intercept giving the value of linear term *χ*_1_. For a better visualization of the fitting, *M*/*H* versus *H*^2^ curve at 10 K on the linear scale is shown in the Fig. 4b. The solid red line shows the fit to the Eq. (3) in the *H* range where the curves exhibit quadratic behaviour. Here, we would like to mention that the fitting range is kept same for all temperatures in order to avoid any kind of discrepancy in extracting the parameters. The inset of Fig. 4b depicts the *T* dependent *χ*_*1*_ data obtained from the fitting and is found to be in analogy to that directly observed from the *χ*_*DC*_ measurements. Both curves follow paramagnetic behaviour down to 1.8 K and do not exhibit any kind of anomaly. Further, Fig. 4c and d show the non-linear terms associated with higher order moments i.e., *χ*_3_ and *χ*_5_ as a function of temperature, respectively. From Fig. 4c, it is noted that *χ*_3_ remains negative for the whole *T* regime (1.8–20) K. This kind of behaviour is reported to arise due to the negative curvature of the Brillouin function in the finite fields^36^ and demands further attention through some microscopic probes. On the other hand, the *χ*_5_ is found to be positive at 1.8 K. On decreasing the *T*, the magnitude of *χ*_5_ shows a decrement with a crossover to negative value around 18 K. The observed behaviour of *χ*_*5*_ can be understood on the basis of the fact that below *T*_*D*_, due to JT distortion, the lowest two singlets are treated as a non-Kramer *S* = $${\raise0.7ex\hbox{$1$} \!\mathord{\left/ {\vphantom {1 2}}\right.\kern-0pt} \!\lower0.7ex\hbox{$2$}}$$ pseudo-doublet separated by *δ* ~ 1.3 K^29^ and thus, Tb^3+^ ion behaves as a pseudospin—$${\raise0.7ex\hbox{$1$} \!\mathord{\left/ {\vphantom {1 2}}\right.\kern-0pt} \!\lower0.7ex\hbox{$2$}}$$ magnetic ion at low temperatures. This pseudospin—$${\raise0.7ex\hbox{$1$} \!\mathord{\left/ {\vphantom {1 2}}\right.\kern-0pt} \!\lower0.7ex\hbox{$2$}}$$ Tb^3+^ magnetic ion is believed to carry the higher order moments, and the interactions among these magnetic ions tends to stabilize the higher order moments phase^39,40^. To understand this development of new magnetic phase associated with positive *χ*_5_, a two-level system having an energy splitting of *δ* should be adequate enough^41,42^. Assuming that the magnetic field is parallel to the quantization axis and the quantum spins are treated as discrete, the linear and non-linear terms can be described as^42^:

4 $$\chi_{1} = \frac{{\gamma^{2} }}{\delta }\frac{1}{\tau }\frac{1}{1 + A}$$

5 $$\chi_{3} = \frac{{\gamma^{4} }}{{3!\delta^{3} }}\frac{1}{{\tau^{3} }}\frac{A - 2}{{\left( {1 + A} \right)^{2} }}$$

6 $$\chi_{5} = \frac{{\gamma^{6} }}{{5!\delta^{5} }}\frac{1}{{\tau^{5} }}\frac{{A^{2} - 13A + 16}}{{\left( {1 + A} \right)^{3} }}$$

where *A* = 0.5 e^1/τ^ and τ = *k*_B_*T*/*δ*. It is clearly visible from the Eq. (5) that *χ*_*3*_ = 0 for *A* = 2 and *χ*_3_ is negative for *A* < 2. As seen from the Fig. 4c, *χ*_3_ remains negative in the *T* range of measurement, implying that *A* should always be less than 2. Similarly, the solution of Eq. (6) indicates that *χ*_5_ < 0 for 1.38 < *A* < 11.63. As the crossover from negative to positive value for *χ*_5_ occurs at 18 K, which is possible for either *A* < 1.38 or *A* > 11.63. Thus, from the above analysis, it can be inferred that *A* should always be less than 1.38. The value of the splitting *δ* can be calculated using *T* (~ 18 K) at which *χ*_5_ ~ 0 and is found to be 18.3 K. In this state (i.e., above 10 kOe), it is believed that the out-of-plane (or perpendicular) component of magnetic anisotropy exhibits magnetic field dependence and becomes dominating at higher fields. As a consequence of this, the Zeeman energy related with the magnetic anisotropy becomes significantly important, resulting in an increment in the splitting *δ* on application of magnetic field. This enhanced separation is believed to be responsible for the evolution of this new magnetic phase of higher order moments^40^. In order to support our experimental observation, we have used a theoretical model which calculates the *δ* as a function of magnetic field^43^, discussed in the next section.

Furthermore, to check the stability of the field induced magnetic phase, magnetic free energy *F* is studied which can be written in the term of *H* and *M* up to sixth order^42^.

7 $$F = - HM + {\text{a}}_{{2}} M^{{2}} + {\text{a}}_{{4}} M^{{4}} + {\text{a}}_{{6}} M^{{6}}$$

In this expansion, the coefficients a_2_, a_4_ and a_6_ are defined as:

8 $${\text{a}}_{{2}} = {1}/{2}\chi_{1}$$

9 $${\text{a}}_{{4}} = - \chi_{3} /{4}\chi_{1}^{4}$$

10 $${\text{a}}_{{6}} = {{\left[ {{3}\chi_{3}^{2} - \chi_{5} \chi_{1} } \right]} \mathord{\left/ {\vphantom {{\left[ {{3}\chi_{3}^{2} - \chi_{5} \chi_{1} } \right]} {\chi_{1}^{7} }}} \right. \kern-0pt} {\chi_{1}^{7} }}$$

It is clearly evident that a_2_ is positive, since *χ*_1_ > 0. Since *χ*_3_ is negative throughout the whole temperature range, implying that a_4_ is also positive. This suggests continuous phase transition. Further, the inset of Fig. 4d shows the plot of temperature dependent a_6_. It is observed that a_6_ remains positive in the entire temperature range of (1.8–20) K. This signifies that free energy is bounded and enters a stable phase transition ascribed to positive fifth -order susceptibility.

## Discussion

**Figure 5:**
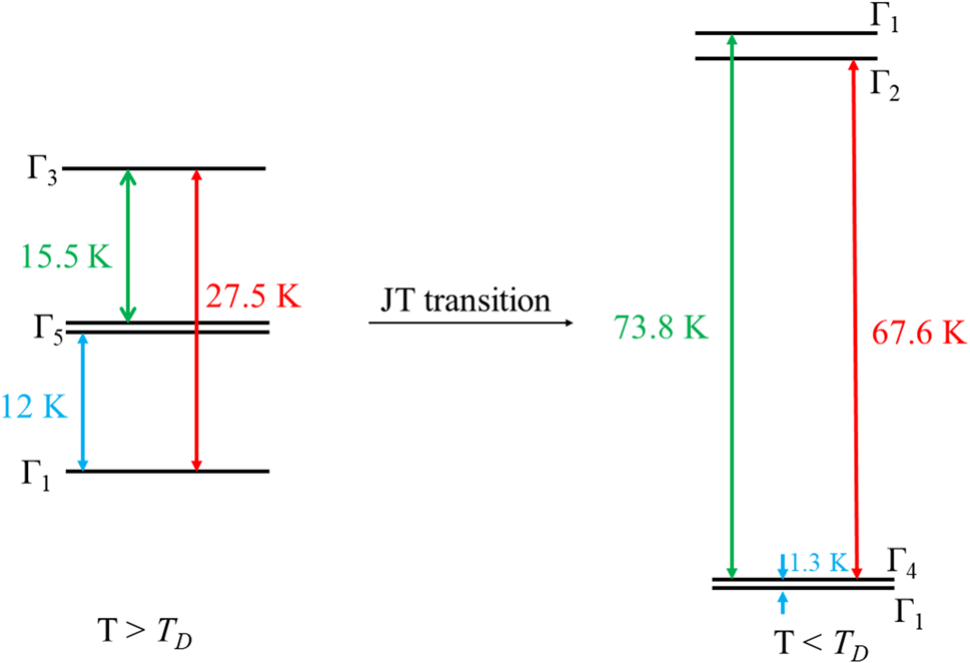
Energy level diagram for TbVO_4_ below and above the structural transition temperature.

As discussed earlier, TbVO_4_ crystallizes in tetragonal structure, in which Tb^3+^ ions are surrounded by eight oxygen ions. These oxygen ions create tetragonal crystal field around Tb^3+^ ion with D_2d_ symmetry. From the ground state term symbol ^7^F_6_ for Tb^3+^ ions, the crystal field splits the lowest manifold J = 6 into (2 J + 1 = 13) crystal field levels. These levels are comprised of three doublets and seven singlets. The lowest four levels consist of two singlets and one doublet. The ground state is a singlet (Γ_1_), followed by a non-Kramer doublet (Γ_5_) at about 12 K and then another singlet (Γ_3_) at around 27.5 K^26,27^. For a better visualization, we have made an energy level diagram shown in the Fig. 5. Further, the structural transition (as also evident from the Fig. 3a) caused by the JT effect at 31 K brings significant changes in the energy of these four levels. The energy of Γ_3_ singlet is increased to 73.8 K, becoming a Γ_1_ singlet. While, the Γ_5_ doublet is splitted into two levels of different energies, one of which is raised in energy by 67.6 K and other is reduced to become Γ_5_, lying just above the ground state Γ_1_ singlet^27–29^. These changes in the energy levels can be more clearly understood from the energy level diagram (Fig. 5). Consequently, the two lowest singlets are treated as a non-Kramer doublet with *S* = $${\raise0.7ex\hbox{$1$} \!\mathord{\left/ {\vphantom {1 2}}\right.\kern-0pt} \!\lower0.7ex\hbox{$2$}}$$, having a splitting of δ ~ 1.3 K^29^. This separation energy is believed to determine the order of the magnetic moments and is expected to get affected on application of external applied magnetic field (as discussed in the heat capacity section). On application of magnetic field, Zeeman energy comes into play in determining the magnetic state of a system as it brings out changes in the crystal energy levels. As mentioned in the magnetization section and also reported in the reference^31^, TbVO_4_ possesses strong magnetic anisotropy with a (= b) axis (a-b basal plane) being the easy axis and c-axis being the hard axis. The anisotropic nature of the ground state is characterized by the values of the anisotropy tensors ($$g_{||}$$ and $$g_{ \bot }$$). Here, $$g_{||}$$ and $$g_{ \bot }$$ are in-plane and out-of-plane components, which are along and perpendicular to the easy axis (a—axis) of the magnetization, respectively. Irrespective of the orientation of the magnetic field, at zero/low field, the ground state of TbVO_4_ exhibits a large magnetic anisotropy with a large value of $$g_{||}$$ as compare to the perpendicular component $$g_{ \bot }$$^31^. For higher magnetic fields, it is believed that the $$g_{ \bot }$$—tensor starts dominating and show a magnetic field dependence. This leads to the enhanced separation of the *δ* associated with pseudospin—$${\raise0.7ex\hbox{$1$} \!\mathord{\left/ {\vphantom {1 2}}\right.\kern-0pt} \!\lower0.7ex\hbox{$2$}}$$ doublet. As a result, the wave-functions of the pseudo-spins can transfer the ground state in time-reversal even or time reversal odd higher-order moments^44,45^. Hence, it can be inferred that at higher magnetic fields, Zeeman effect comes into play resulting in an increment in the splitting of the doublet ground state, which in turn, is believed to be responsible for the development of higher order moments at low temperatures.

Furthermore, we have used a theoretical model^43^ to support our experimental observations. Mainly, our model emphasizes on two aspects: (i) Contribution of out-of-plane component of g-tensor ($$g_{ \bot }$$) at higher fields. (ii) Calculating the *δ* as a function of applied magnetic field. As mentioned earlier, due to JT distortion, the two lowest singlets are treated as pseudospin—$${\raise0.7ex\hbox{$1$} \!\mathord{\left/ {\vphantom {1 2}}\right.\kern-0pt} \!\lower0.7ex\hbox{$2$}}$$ Kramer doublet and the ground state wavefunctions for this can be denoted by $$| \pm$$. The splitting between this ground state doublet simply corresponds to the difference between energy eigen values of doublet wave functions $$| \pm$$ and is equivalent to *δ* ~ 1.3 K at zero field^29^. Therefore, while constructing the model, a few things are taken into account which are mentioned below:

(i) Both anisotropic $g_{||}$ and $g_{ \bot }$ are considered while defining the Hamiltonian to take into account the effect of polycrystallinity on the magnetization data.
(ii) Zero-field splitting of the pseudo-doublet ground state.
(iii) Powder averaging effect.

Thus, the modified Zeeman Hamiltonian is used which is given below in the matrix form as:

11 $$H_{Z}^{^{\prime}} = \left[ {\begin{array}{*{20}c} { - \frac{1}{2}g_{||} {\text{H}}\mu_{B} \cos \theta + \frac{\delta }{2}} & { - \frac{1}{2}g_{ \bot } {\text{H}}\mu_{B} \cos \theta e^{ - i\phi } } \\ { - \frac{1}{2}g_{ \bot } {\text{H}}\mu_{B} \cos \theta e^{i\phi } } & {\frac{1}{2}g_{||} {\text{H}}\mu_{B} \cos \theta - \frac{\delta }{2}} \\ \end{array} } \right]$$

Here, *δ* takes into account the splitting. The other terms account for the Zeeman Hamiltonian on application of magnetic field. Thus, the difference between the energy eigenvalues (*E*_1_ and *E*_2_) for the above Hamiltonian yields the value of *δ* as a function of applied *H* i. e., *δ* (*H*). The terms $g_{||}$ and $g_{ \bot }$ are considered in the Hamiltonian in order to include the effect of polycrystallinity on the measured magnetization.

To find the value of *δ* (*H*), the values of $g_{||}$ and $g_{ \bot }$ are required. These values can be evaluated by fitting the *M* (*H*) data with an equation obtained from the modified Zeeman Hamiltonian (11). Average magnetization $M$, can be calculated by using a very generalized expression given as:

12 $$M = \frac{{m_{1} e^{{ - E_{1} /k_{B} T}} + m_{2} e^{{ - E_{2} /k_{B} T}} }}{{e^{{ - E_{1} /k_{B} T}} + e^{{ - E_{2} /k_{B} T}} }}$$

Here, $m_{1} = - \frac{{\partial E_{1} }}{\partial H}$ and $m_{2} = - \frac{{\partial E_{2} }}{\partial H}$. *E*_1_ and *E*_2_ corresponds to the energy eigenvalues obtained by solving the Hamiltonian described in the Eq. (11). Considering only the easy axis ($g_{||}$) (i.e., $g_{ \bot }$ = 0) and *δ* = 0, the above equation reduces to the standard equation described in^47^:

13 $$M = \frac{1}{2}g_{||} \mu_{B} \cos \theta \tan {\text{h}}\left( {\frac{{g_{||} {\text{H}}\mu_{B} \cos \theta }}{{2k_{B} T}}} \right)$$

Further, the integration of the Eq. (12) with the consideration of all possible orientations gives the expression for $M$ which is given as:

14 $$M = \frac{1}{4\pi }\int {\mathop \int \limits_{0}^{\pi } \mathop \int \limits_{0}^{2\pi } } \frac{{m_{1} e^{{ - E_{1} /k_{B} T}} + m_{2} e^{{ - E_{2} /k_{B} T}} }}{{e^{{ - E_{1} /k_{B} T}} + e^{{ - E_{2} /k_{B} T}} }} \sin \theta d\theta d\phi$$

**Figure 6:**
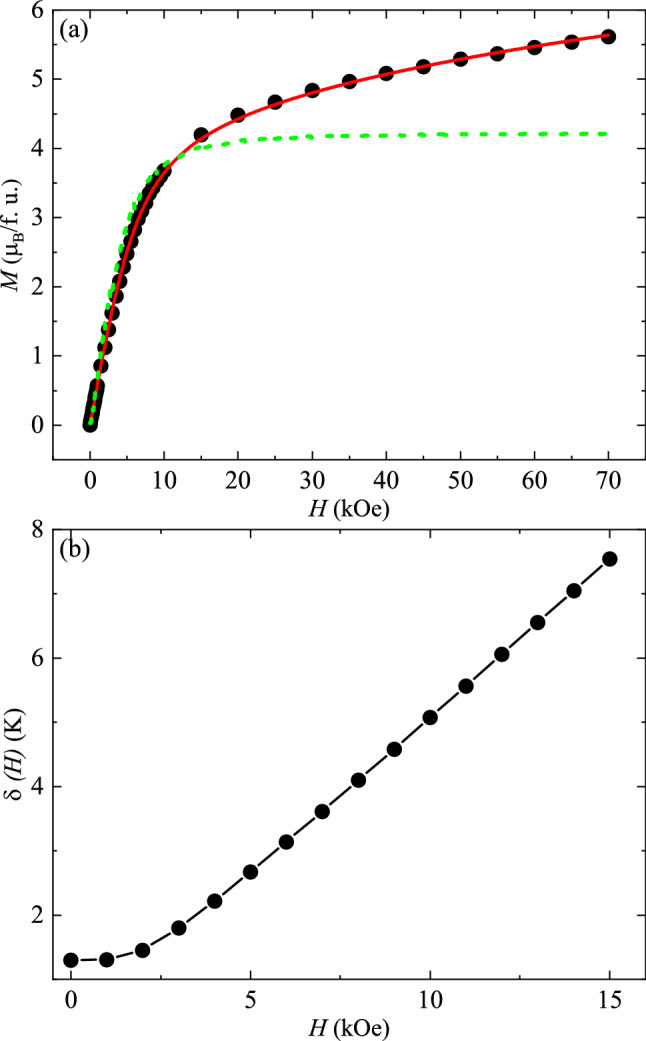
(**a**) *M* (*H*) curve obtained at 2 K. The dash green and solid red lines show the fit to the Eqs. (13) and (14), respectively. (**b**) The splitting of the pseudo-doublet ground state (*δ*) as a function of applied *H* calculated by solving the Eq. (15).

Afterward, in order to investigate the role of $g_{ \bot }$ at higher fields, we have first fitted the experimentally obtained *M* (*H*) data by considering the limiting case as described in the Eq. (13). The dash green line in the Fig. 6a shows the fitting to the Eq. (13) with the fact that the zero-field splitting (*δ*) is also taken into account. The obtained parameters are $g_{||}$ ~ (16.91 ± 0.08) and $g_{ \bot }$ = 0. From the figure, it is clearly visible that the data fits very well up to 10 kOe and then shows strong deviation. This implies that at higher fields, our assumption of considering only easy-axis fails and the out-of-plane component of anisotropy plays an important role at fields above 10 kOe.

Thus, it will be appropriate to take $g_{ \bot }$ as a function of *H*. In order to investigate the *H*-dependence of $g_{ \bot }$, we have tried different combinations and it is concluded that $g_{ \bot }$ ~ *H*^0.1^ dependence shows the best match with the experimental data (shown by the solid red line in Fig. 6a). The values of the parameters $g_{||}$ and $g_{ \bot }$ from the fitting are found to be (14.30 $\pm$ 0.02) and ((3.78 $\pm$ 0.01) $\times$ *H*^0.1^), respectively. From here, it can be concluded that the $g_{ \bot }$ tensor increases with field and becomes more dominating at higher fields, which in turn is expected to increase the splitting of the pseudo-doublet at higher fields.

Afterward, *δ* (*H*) is evaluated by integrating the energy difference (*E*_*2*_ − *E*_*1*_) with respect to all possible orientations and is given by:

15 $$k_{B} \delta \left( H \right) = \frac{1}{4\pi }\int {\mathop \int \limits_{0}^{\pi } \mathop \int \limits_{0}^{2\pi } } 2\left( {\frac{{g_{||}^{2} H^{2} \mu_{B}^{2} }}{2}cos^{2} \theta + \frac{{g_{ \bot }^{2} H^{2} \mu_{B}^{2} }}{2}sin^{2} \theta + \frac{{\delta^{2} }}{4} - \frac{{g_{||} H\mu_{B} }}{2}\delta sin\theta } \right)^{1/2} \sin \theta d\theta d\phi$$

Figure 6b represents the *H* dependent $$\delta$$ (in units of K), calculated by solving the Eq. (15). It shows that the $$\delta$$ increases linearly on increasing the magnetic field. So, our experimental as well as theoretical results indicate towards an increment of the splitting of the pseudospin—$${\raise0.7ex\hbox{$1$} \!\mathord{\left/ {\vphantom {1 2}}\right.\kern-0pt} \!\lower0.7ex\hbox{$2$}}$$ doublet on application of external *H*. From here, it can be inferred that on increasing the external magnetic field, the out-of-plane component of the magnetic anisotropy ($$g_{ \bot }$$) starts dominating. This brings Zeeman effect into act and results in the enhanced splitting ($$\delta$$) of the pseudo-doublet ground state. This enhanced $$\delta$$ is further believed to stabilize the exotic magnetic phase associated with positive *χ*_5_ at higher fields and low temperatures.

Finally, we would like to mention that Kazei et al., have performed field dependent magnetic measurements up to a field of 500 kOe, along with crystal field calculations, on TbVO_4_^47^. According to their report, a quadrupole ordering takes place at the structural transition temperature (~ 31 K). Application of magnetic field destroys this ordering at a critical field of 320 kOe and the system again transforms into tetragonal structure from the orthorhombic structure. Our study also focuses on the consequence of the external field on the JT driven structural transition. Our results reveal that a field induced magnetic phase associated with fifth order susceptibility develops in the orthorhombic phase (below 20 K); as a consequence of enhanced splitting between the lowest two levels.

## Conclusion

In this work, we have investigated the effect of external magnetic field on the magnetic ground state of TbVO_4_ through the means of field dependent magnetization, heat capacity and non-linear DC susceptibility, along with a theoretical model. JT distortion below 31 K results in the formation pseudospin—$${\raise0.7ex\hbox{$1$} \!\mathord{\left/ {\vphantom {1 2}}\right.\kern-0pt} \!\lower0.7ex\hbox{$2$}}$$ doublet ground state with splitting *δ* ~ 1.3 K. Field dependent magnetization shows a slope change around 10 kOe. Above this field, Zeeman energy linked with the magnetic anisotropy results in an enhancement in the splitting *δ*. This is further supported by our theoretical model which points that *δ* increases with increments in *H*. This enhanced splitting further stabilizes the magnetic phase associated with higher order moments. Our investigation may provide a pathway to researchers for exploring the magnetic ground state of JT systems in presence of magnetic field through microscopic experimental probes or theoretical studies.

## Methods

Polycrystalline sample of TbVO_4_ was synthesised by the conventional solid state reaction method by using high purity Tb_2_O_3_ and VO_2_ from Sigma Aldrich with 99.9% purity. The initial materials in stochiometric ratio were grinded and given heat treatment at 800 °C. The obtained product was palettized and sintered at 800 °C. Room temperature powder XRD was performed in the range (10°–90°) using Rigaku diffraction with Cu Kα (λ = 1.54) radiation. The crystal structure was refined by the Rietveld method using the FullProf Suite software. Magnetic field and temperature dependent magnetic measurements in the temperature range 1.8–300 K were performed using the Magnetic Property Measurement System (MPMS) from Quantum Design, USA. Physical Property Measurement System (PPMS), Quantum Design, USA was used to measure temperature dependent heat capacity at different fields up to 70 kOe.

## Data Availability

All the data generated and/or analysed during the current study are included in the published article.
